# Identification of areas of grading difficulties in prostate cancer and comparison with artificial intelligence assisted grading

**DOI:** 10.1007/s00428-020-02858-w

**Published:** 2020-06-15

**Authors:** Lars Egevad, Daniela Swanberg, Brett Delahunt, Peter Ström, Kimmo Kartasalo, Henrik Olsson, Dan M. Berney, David G. Bostwick, Andrew J. Evans, Peter A. Humphrey, Kenneth A. Iczkowski, James G. Kench, Glen Kristiansen, Katia R. M. Leite, Jesse K. McKenney, Jon Oxley, Chin-Chen Pan, Hemamali Samaratunga, John R. Srigley, Hiroyuki Takahashi, Toyonori Tsuzuki, Theo van der Kwast, Murali Varma, Ming Zhou, Mark Clements, Martin Eklund

**Affiliations:** 1 Department of Oncology-Pathology, Karolinska Institutet|, Karolinska University Hospital, Radiumhemmet P1:02, 171 76 Stockholm, Sweden; 2 Department of Pathology and Molecular Medicine, Wellington School of Medicine and Health sciences, University of Otago, Wellington, New Zealand; 3 Department of Medical Epidemiology and Biostatistics, Karolinska Institutet, Stockholm, Sweden; 4 Barts Cancer Institute, Queen Mary University of London, London, UK; 5 Bostwick Laboratories, Orlando, FL USA; 6 Laboratory Medicine Program, University Health Network, Toronto, ON Canada; 7 Department of Pathology, Yale University School of Medicine, New Haven, CT USA; 8 Department of Pathology, Medical College of Wisconsin, Milwaukee, WI USA; 9 Department of Tissue Pathology and Diagnostic Oncology, Royal Prince Alfred Hospital and Central Clinical School, University of Sydney, Sydney, New South Wales Australia; 10 Institute of Pathology, University Hospital Bonn, Bonn, Germany; 11 Department of Urology, Laboratory of Medical Research, University of Sao Paulo Medical School, Sao Paulo, Brazil; 12 Cleveland Clinic, Pathology and Laboratory Medicine Institute, Cleveland, OH USA; 13 Department of Cellular Pathology, Southmead Hospital, Bristol, UK; 14 Department of Pathology, Taipei Veterans General Hospital, Taipei, Taiwan; 15 Aquesta Uropathology and University of Queensland, Brisbane, Queensland Australia; 16 Department of Laboratory Medicine and Pathobiology, University of Toronto, Toronto, ON Canada; 17 Department of Pathology, The Jikei University School of Medicine, Tokyo, Japan; 18 Department of Surgical Pathology, School of Medicine, Aichi Medical University, Nagoya, Japan; 19 Department of Cellular Pathology, University Hospital of Wales, Cardiff, UK; 20 Department of Pathology and Laboratory Medicine, Tufts Medical Center and Tufts School of Medicine, Boston, MA USA

**Keywords:** Pathology, Standardization, Grading, Reproducibility, Artificial intelligence, Prostate cancer

## Abstract

The International Society of Urological Pathology (ISUP) hosts a reference image database supervised by experts with the purpose of establishing an international standard in prostate cancer grading. Here, we aimed to identify areas of grading difficulties and compare the results with those obtained from an artificial intelligence system trained in grading. In a series of 87 needle biopsies of cancers selected to include problematic cases, experts failed to reach a 2/3 consensus in 41.4% (36/87). Among consensus and non-consensus cases, the weighted kappa was 0.77 (range 0.68–0.84) and 0.50 (range 0.40–0.57), respectively. Among the non-consensus cases, four main causes of disagreement were identified: the distinction between Gleason score 3 + 3 with tangential cutting artifacts vs. Gleason score 3 + 4 with poorly formed or fused glands (13 cases), Gleason score 3 + 4 vs. 4 + 3 (7 cases), Gleason score 4 + 3 vs. 4 + 4 (8 cases) and the identification of a small component of Gleason pattern 5 (6 cases). The AI system obtained a weighted kappa value of 0.53 among the non-consensus cases, placing it as the observer with the sixth best reproducibility out of a total of 24. AI may serve as a decision support and decrease inter-observer variability by its ability to make consistent decisions. The grading of these cancer patterns that best predicts outcome and guides treatment warrants further clinical and genetic studies. Results of such investigations should be used to improve calibration of AI systems.

## Background

The Gleason grading system was introduced more than half a century ago but is still one of the most powerful prognostic and predictive factors for prostate cancer. One of the strengths of Gleason grading is that it takes into account the striking heterogeneity seen in cancers of the prostate. Nevertheless, the reporting of this morphological information remains a challenge as it requires both the classification of patterns and the estimation of their extent. Similar to other semi-quantitative data in pathology, Gleason grading suffers from inter-observer variability [1]. This is not surprising since prostate cancer grading assesses complex morphological patterns that are estimated visually and translated into an ordinal scale. Despite numerous efforts to reach consensus, the boundaries between the grades remain subjective [2–4]. In addition, the definitions of morphological patterns and the rules for the calculation of the Gleason score have been revised several times leading to variations in the interpretation of grading criteria [5, 6].

In an attempt to standardize grading, the International Society of Urological Pathology (ISUP) launched a reference image database known as Pathology Imagebase. This online tool is supervised by expert pathology sub-specialists, with the purpose of establishing an international standard in prostate cancer grading [3, 7]. This has resulted in the achievement of expert consensus in many of the database cases. Despite this, the expert panel failed to agree on a substantial subset of cases indicating a need for further standardization.

There is now a considerable interest in the use of artificial intelligence (AI) for the development of qualified decisions in clinical medicine, including the reporting of pathology specimens [8, 9]. A strength of AI is its ability to process data rapidly and in a consistent manner. We have recently developed an AI system for the detection and grading of prostate cancer in needle biopsies [10]. Validation studies were undertaken as part of this development, including assessments on independent and external test sets, and here the system was remarkably precise in both diagnosis and grading. When applied to the ISUP Imagebase cases, the performance of the AI tool was within the range of the results of the expert panel. [10]

The focus of this study was an analysis of the Imagebase cases that did not reach consensus. In particular, we aimed to compare expert grading with AI-assisted grading and analyze the nature and causes of grading disagreement.

## Materials and methods

Pathology Imagebase is hosted on the ISUP Web site, and prostate cancer cases have been uploaded and graded by leading experts [3, 7]. A group of 23 internationally acknowledged experts in urological pathology representing geographic regions from around the world submitted complete voting on all study cases that had been loaded onto the Web site. For these cases, the Gleason score options were 3 + 3 = 6, 3 + 4 = 7, 4 + 3 = 7, 4 + 4 = 8, and 9–10 (also known as ISUP grades 1–5) and Other (specified). Consensus was defined as 16 votes in favor of a single diagnostic option, which corresponded to an agreement by two-thirds of the panel. Consensus cases are available in a public database domain for education purposes and to promote the international standardization of grading. Specifically, the expert panel independently reviewed microphotographs of 90 cases of needle biopsies containing prostate cancer that had been uploaded between May and September 2015. Each image was obtained from a single biopsy core from the Stockholm 3 (STHLM3) study, a population-based screening study undertaken among men aged 50–69 years [11]. Microphotographs were taken by a digital camera (SPOT Imaging, Sterling Heights, MI, USA) at 2048 × 2048 pixels. For publication online, the resolution was reduced to 72 dpi. The Imagebase Web site can be accessed by any standard Web browser and viewed on standard screens. How it was accessed was not controlled for. There was an overrepresentation of high-grade cancers among the uploaded images and these were included to represent different morphologies and challenging cases.

Glass slides of 87 of the 90 biopsies were available for scanning and AI analysis. Slides were scanned using an Aperio ScanScope AT2 scanner and Aperio Image Library v. 12.0.15 software (Leica Biosystems, Wetzlar, Germany). The scanned images were processed by AI as previously described [10]. The AI system consisted of two convolutional deep neural network ensembles, each consisting of 30 Inception V3 models pre-trained on ImageNet, with classification layers adapted to our outcome [10]. The system was trained on 6682 digitized needle biopsies from 976 randomly selected participants in the STHLM3 study conducted between May 28, 2012, and December 30, 2014, and subsequently evaluated by predicting the presence, extent, and Gleason grade of malignant tissue for independent and external test sets comprising 1961 biopsies from 218 men [10, 11].

The results of these cases were further analyzed in this study. None of the 87 cases had been part of the dataset used for training or validation of AI. All non-consensus cases in the database were reviewed by two of the authors (D.S., L.E.), and discrepancies in grading were identified and classified in categories.

Weighted kappa values for multiple observers were calculated using O’Connell and Dobson estimators [12]. The average agreement for each case was assessed using linear weights. The mean weighted kappas by a pathologist were calculated using Schouten’s methodology [13]. To consider agreement for a specific grade, we dichotomized the results and used unweighted kappas. All the kappas were calculated using the Magree package in R [14]. Bootstrap was used for computing of 95% confidence intervals.

## Results

**Fig. 1:**
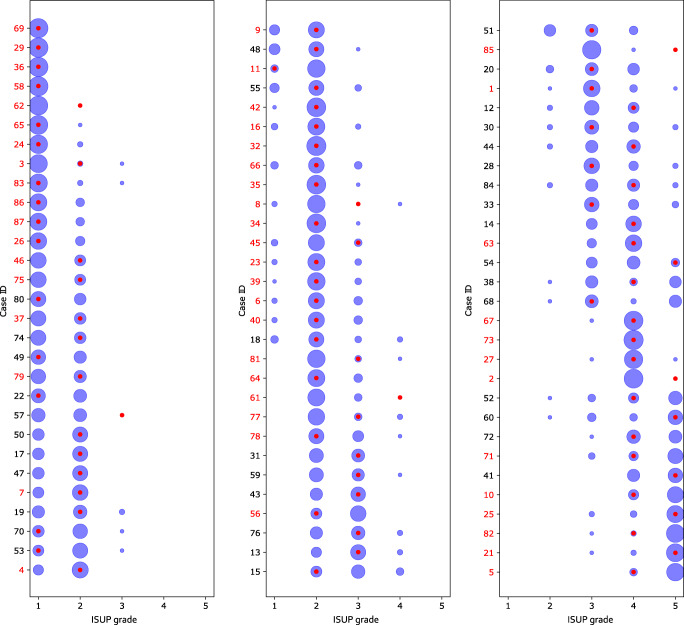
Grading performance relative to ISUP expert panel on Imagebase. The distribution of ISUP scores given by the 23 pathologists from the ISUP expert panel and the AI for each of the 87 case IDs in Imagebase. Each row corresponds to one case, and the cases are organized into three plots according to average ISUP score increasing from left to right, and from top to bottom. The areas of the blue circles represent the proportion of pathologists who voted for a specific ISUP score (*x*-axis). The red dot indicates the ISUP score given by the AI. Example: In the last row (bottom-right; case ID 5) most pathologists voted ISUP 5 and a minority ISUP 4; the red dot indicates that AI voted ISUP 4

All 90 cases were graded by 23 panel members. Among the 87 cases with slides available for AI analysis, a 2/3 consensus grade was reached in 58.6% (51/87) of cases, while 41.4% (36/87) failed to reach consensus. The distribution of the grades assigned by the panel members is illustrated by the size of the blue circles in Fig. 1, while the AI grades are marked with red dots. The panel members assigned a Gleason score of 3 + 3 = 6, 3 + 4 = 7, 4 + 3 = 7, 4 + 4 = 8, and 9–10 in 17.0% (147), 30.8% (266), 25.9% (224), 15.5% (134), and 10.3% (89), respectively, and Other in 0.5% (one vote of 2 + 3 and three votes of 3 + 5). The AI system assigned a Gleason score of 3 + 3 = 6, 3 + 4 = 7, 4 + 3 = 7, 4 + 4 = 8, and 9–10 in 13.9% (5), 25.0% (9), 33.3% (12), 19.4% (7), and 8.3% (3), respectively. The AI grades were the same as the majority vote in 61.1% (22/36) of non-consensus cases. In 6 non-consensus cases, the AI grade was lower than the mode of the expert grades and in 8 cases it was higher.

The best overall agreement between the pathologists and the AI system was achieved in cases that were assigned a Gleason score of 3 + 3 by the panel members (72%), while the lowest agreement was achieved for Gleason score 4 + 3 cancers (38.6%) (Tables 1 and 2). Between the pathologists alone the best agreement was reached for Gleason score 3 + 3 cancers (70.6%) and the worst for Gleason score 4 + 3 (44.4%) (Tables 1 and 2).

**Table 1:** Agreement (%) between AI grades and the pathologists’ grading by ISUP grade (all cases)

	ISUP grades assigned by Imagebase panel (%)				
ISUP grades by AI	1	2	3	4	5
1	72.0	27.2	0.8	0	0
2	29.7	58.8	10.5	1.0	0
3	4.3	39.9	38.6	12.5	4.6
4	0	7.3	19.8	43.5	29.3
5	0	0.6	23.6	33.5	42.2

**Table 2:** Average and range agreement (%, mean, range) across all pathologists by ISUP grade (all cases)

	ISUP grades assigned by other pathologists in the Imagebase panel (%, mean, range)				
ISUP grade by pathologist	1	2	3	4	5
1	70.6 (56.7–86.4)	27.9 (13.3–40.2)	1.4 (0.3–3.1)	0.1 (0–0.5)	0
2	20.7 (8.9–38.4)	63.5 (56.6–72.3)	13.1 (4.8–23.2)	2.3 (0.2–6.2)	0.3 (0–3.1)
3	2.4 (0–13.1)	27.0 (4.2–60.5)	44.4 (26.4–55.6)	18.6 (2.8–42.0)	7.6 (0.3–23.6)
4	0.1 (0–1.4)	4.8 (0–21.9)	20.9 (1.8–37.7)	58.5 (41.1–80.0)	15.6 (0.8–34.5)
5	0	1.0 (0–2.1)	12.3 (2.3–21.8)	20.3 (11.4–29.9)	66.4 (52.9–86.4)

**Fig. 2:**
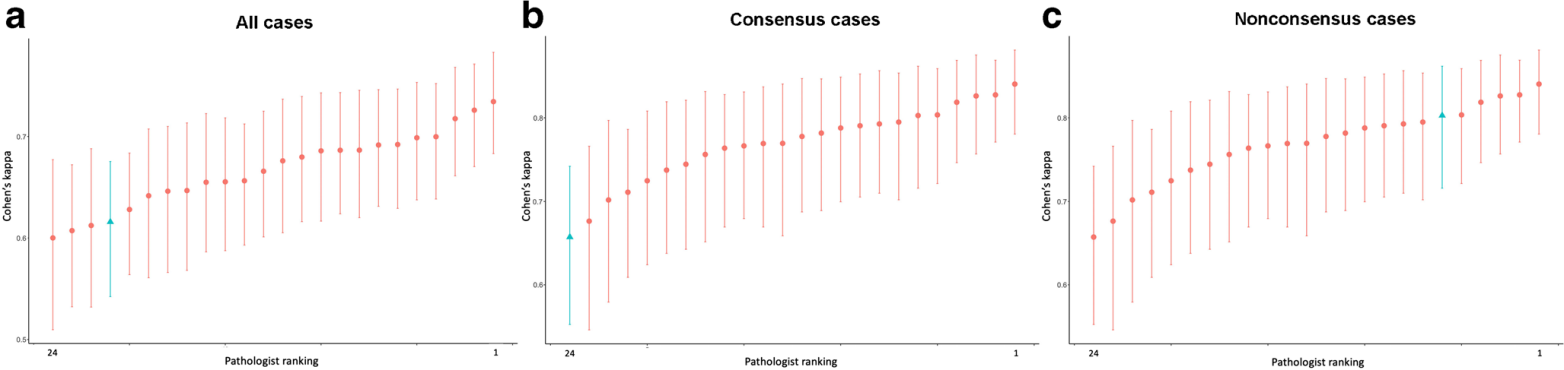
**a**–**c** Mean weighted kappas for International Society of Urological Pathology (ISUP) grades 1–5 of 24 observers with complete data submission for all cases (**a**), consensus cases (**b**), and non-consensus cases (**c**). The AI system’s mean kappa is shown as a triangle in blue color, and the 23 expert pathologists in the Imagebase reference panel as red dots. Whiskers indicate 95% confidence intervals

The mean weighted kappas for all cases, the consensus cases and the non-consensus cases were 0.67 (range 0.60–0.73), 0.77 (range 0.68–0.84), and 0.50 (range 0.40–0.57), respectively. The weighted kappas of the AI system against the observers for all cases, the consensus cases and the non-consensus cases were 0.63, 0.66 and 0.53, respectively. In Fig. 2a–c, the kappa statistics of individual observers and the AI system are shown in order of magnitude for all cases, consensus cases and non-consensus cases.

**Fig. 3:**
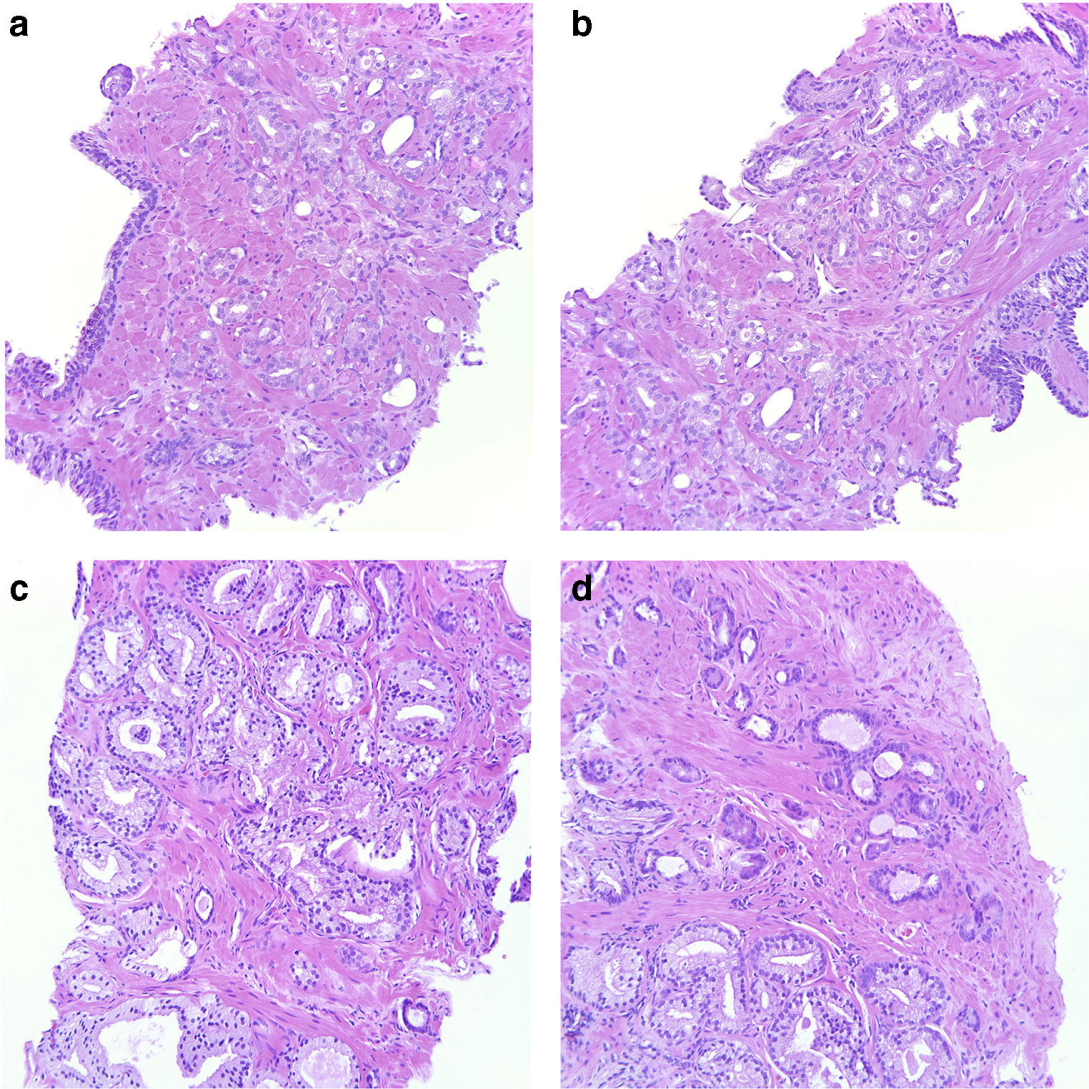
**a**, **b** Cancer bordering between Gleason score 3 + 3 = 6 with tangential cuts and Gleason score 3 + 4 = 7 with poorly formed glands. Panel members voted 3 + 3 = 6 in 54.2% and 3 + 4 = 7 in 45.8% and AI assigned a Gleason score of 3 + 3 = 6. **c**, **d** Cancer bordering between Gleason score 3 + 3 = 6 with tangential cuts and Gleason score 3 + 4 = 7 with fused glands, particularly in **c**. Panel members voted 3 + 3 = 6 in 37.5% and 3 + 4 = 7 in 58.3% and AI assigned a Gleason score of 3 + 4 = 7. All microphotographs show hematoxylin and eosin stains at × 20 lens magnification

Among the non-consensus cases, four main causes of disagreement were identified (Table 3). The most common problem was the distinction between Gleason score 3 + 3 with tangential cutting artifacts vs. Gleason score 3 + 4 tumors with poorly formed or fused glands as seen in 13 cases (10 with only seemingly poorly formed glands, 2 with only seemingly fused glands and 1 with both). In 8 of these cancers, AI opted for the higher grade. Figure 3 a and b show two fields of a case with seemingly poorly formed glands where AI suggested a Gleason score of 3 + 3, and Fig. 3 c and d show two fields of a case with seemingly fused glands where AI suggested a Gleason score of 3 + 4.

**Table 3:** Causes of disagreement between pathologists among non-consensus cases of ISUP Imagebase and results of AI. GS = Gleason score, GP = Gleason pattern

Causes of disagreement	Number of cases	AI results
GS 3 + 3 with tangential cutting artifacts vs. GS 3 + 4 with poorly formed or fused glands	13	3 + 4 in 8/13
GS 3 + 4 vs. 4 + 3	7	4 + 3 in 6/7
GS 4 + 3 vs. 4 + 4	8	4 + 4 in 4/8
Identification of small component of Gleason pattern 5	6	4 + 5/5 + 4 in 2/6
Other (a possible glomeruloid body, mucinous cancer)	2	3 + 3 and 4 + 3
Total non-consensus	36

**Fig. 4:**
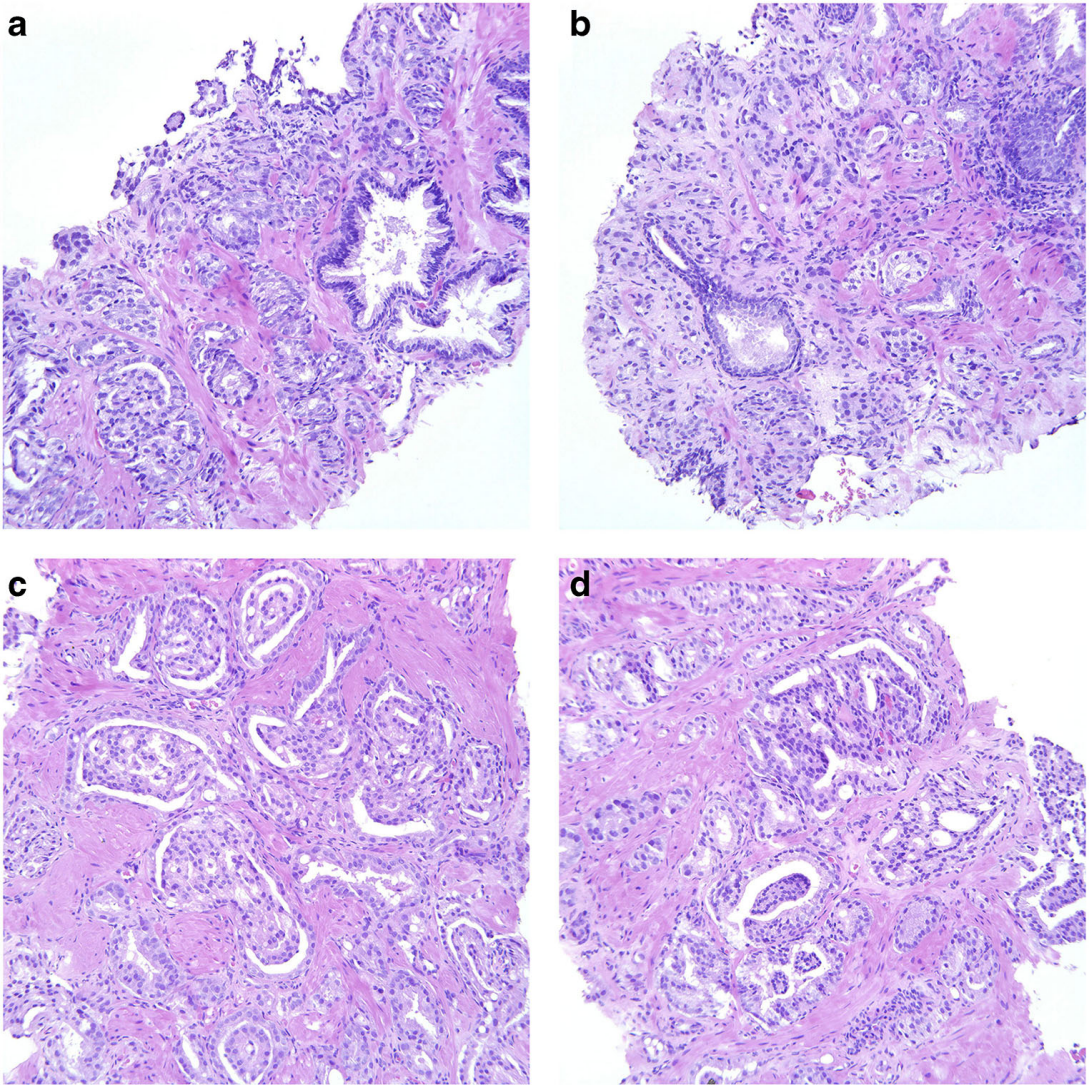
**a**, **b** Cancer bordering between Gleason score 3 + 4 = 7 and 4 + 3 = 7. Panel members voted 3 + 4 = 7 in 33.3% and 4 + 3 = 7 in 58.3% and AI assigned a Gleason score of 4 + 3 = 7. **c**, **d** Cancer bordering between Gleason score 4 + 3 = 7 and 4 + 4 = 8. Mostly cribriform and glomeruloid glands but also occasional separate glands, particularly in **d**. Panel members voted 4 + 3 = 7 in 37.5% and 4 + 4 = 8 in 62.5% and AI assigned a Gleason score of 4 + 4 = 8. All microphotographs show hematoxylin and eosin stains at × 20 lens magnification

The estimation of the proportions of Gleason patterns 3 and 4 in Gleason score 7 cancers, i.e., the distinction between Gleason scores 3 + 4 and 4 + 3 was a cause of disagreement in 7 cases. Here, the AI system assigned the higher grade in 6 cases. Figure 4 a and b illustrate two fields of one of these cases.

In 8 cases, the problem was to determine whether a minor component of Gleason pattern 3, in a cancer dominated by Gleason pattern 4, should be included in the Gleason score resulting in a Gleason score of 4 + 3 or if it could be overlooked. The AI assigned a Gleason score of 4 + 3, 4 + 4, and 4 + 5/5 + 4 in 2, 4, and 1 case, respectively. Figure 4 c and d show two fields of one of the cases where AI opted for Gleason score 4 + 4.

**Fig. 5:**
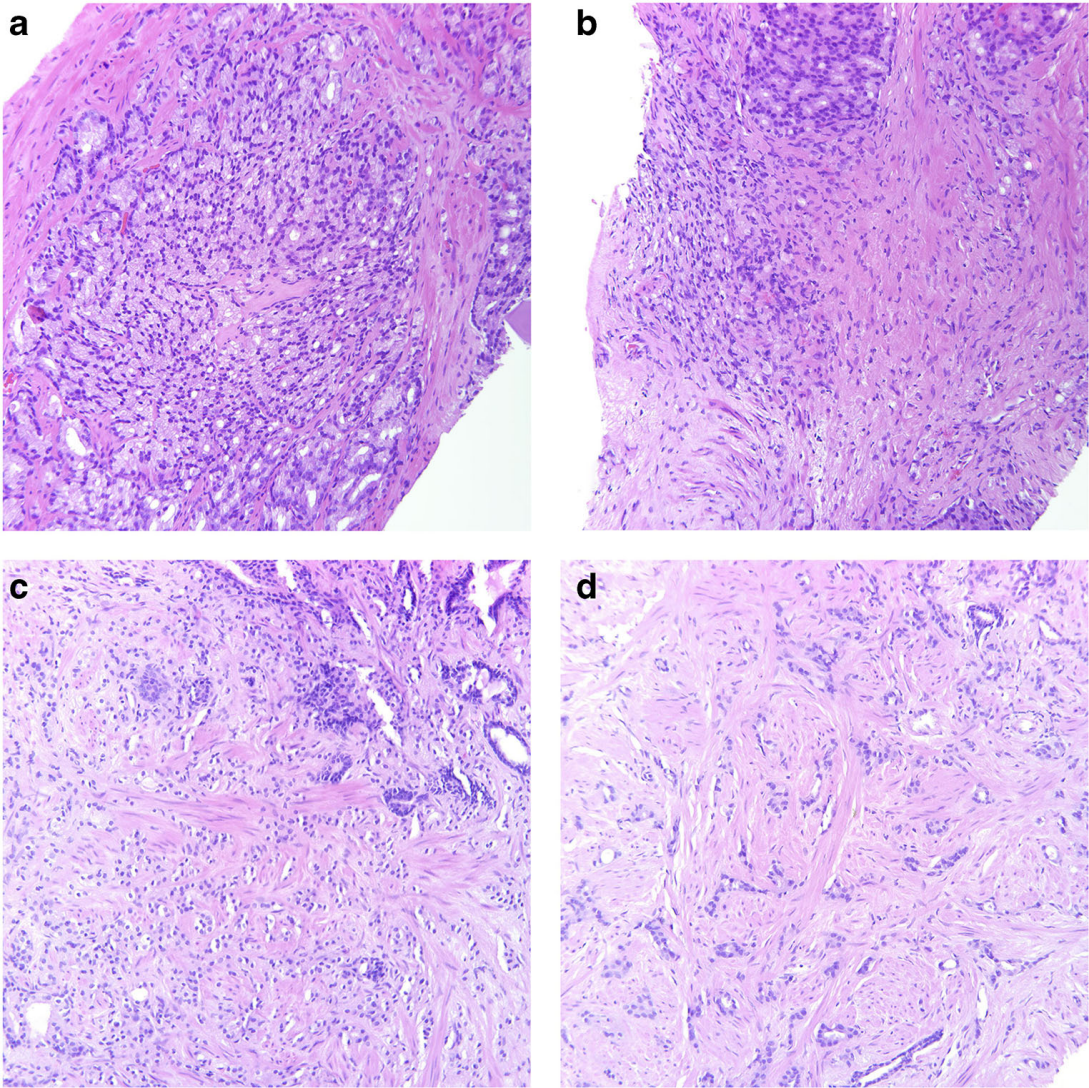
**a**, **b** Cancer bordering between Gleason score 4 + 4 = 8 and Gleason score 9. The tumor is dominated by cribriform cancer but there is also an area with some seemingly dispersed cells and strands, particularly in **b**. The possibility of crush artifacts may be considered. Panel members voted 4 + 4 = 8 in 41.7% and 9 in 58.3% and AI assigned a Gleason score of 9. **c**, **d** Cancer bordering to Gleason score 9. Pale cells forming some gland-like nests but strands and some single cells are also seen, particularly in **c**. Newly diagnosed cancer with no history of hormonal treatment. Poorly formed, tadpole-like structures with tapered and sometimes transitions to strands are seen in **d**. Panel members voted Gleason score 9 in 58.3% while the remainder were spread across Gleason scores 3 + 4, 4 + 3, and 4 + 4. AI assigned a Gleason score of 9. All microphotographs show hematoxylin and eosin stains at × 20 lens magnification

In another 6 cases, the problem was the identification of a small component of Gleason pattern 5 (Fig. 5). Only two of these were acknowledged by the AI as having a Gleason pattern 5 component, illustrated in Fig. 5a, b and Fig. 5c, d, respectively.

In the remaining two non-consensus cases, the problems were the grading of a component of mucinous cancer, which was interpreted as Gleason pattern 4 by AI, and the grading of a possible glomeruloid structure, which was ignored by AI, resulting in a Gleason score of 3 + 3.

In 6 of 87 cases, the AI system suggested grades different from those assigned by any of the experts. This included ISUP grades 2, 3, 4, and 5 in 1, 2, 2, and 1 cases, respectively. On the other hand, the AI system assigned an ISUP grade 5 in two cases where the experts agreed on a consensus diagnosis of ISUP grade 2 and 4, respectively.

## Discussion

The problem with the standardization of grading in pathology is that it relies on the subjective interpretation of a set of rules, which themselves are often unclear. The grading of prostate cancer is probably better defined than that of many other tumors as it contains numerous architectural descriptors such as cribriform glands, glandular fusion or single cell invasion. Conversely, other grading systems are often based on the separation of a continuous range of features into an ordinal scale such as mild, moderate, or severe nuclear atypia. Furthermore, the Gleason grading system only factors in architectural features and does not include other cellular details such as nuclear atypia or mitotic counts. Thus, prostate cancer grading is a monoparametric grading which has the advantage of eliminating conflicts between different features that do not parallel each other. Prostate cancer grading has been the subject of numerous international consensus conferences and the literature on the topic is extensive [5, 6]. Despite these often detailed instructions, a problem with inter-observer variability remains. Countless studies have been conducted on the issue [2–4, 15–17], and they generally show a reproducibility among uropathology experts within the ranges of moderate and substantial (weighted kappa 0.41–0.60 and 0.61–0.80, respectively) [3, 15, 17]. The reproducibility among general pathologists tends to be somewhat lower than that of the experts, usually ranging in the lower end of moderate [2, 16].

Pathology Imagebase is an attempt to establish a publicly available image repository for calibration of grading, with the understanding that there is a requirement for image-based recommendations rather than just a set of written instructions and a schematic diagram [3, 7]. Moreover, Imagebase differs from other Web-based resources as it was generated by a large expert panel consisting of the leaders in the field across the globe. Importantly, these participants have submitted their grading independently, which ensures that the Imagebase is informed by unbiased expert opinions. The focus of the current study relates to the non-consensus cases of Imagebase and an analysis of the causes of disagreement. We also aimed to study the performance of an AI system in problem cases.

While the overall inter-observer agreement in the Imagebase repository was in the range of substantial with a weighted kappa of 0.67, a problematic disagreement remained in some cases, even among these very experienced pathologists who have devoted their careers to urological pathology. In as many as 41.1% of the 87 cases the experts failed to reach a 2/3 agreement. These results are obviously unsatisfactory, although it needs to be emphasized that the disagreement is most likely lower in a routine consecutive series of cases, rather than the current series, which was specifically designed to include problematic higher grade tumors. In a consecutive series there would be a higher proportion of Gleason score 3 + 3 cancers, which are associated with a lower level of inter-observer variability [3].

Although the cancers represented in Imagebase do not encompass all problematic grading scenarios, an analysis of cases that failed to reach consensus did identify a number of problem areas. The most common source of disagreement was the distinction between Gleason score 3 + 3 with tangential cuts and Gleason score 3 + 4 with poorly formed or fused glands. It has been shown that the reproducibility of Gleason pattern 4 with poorly formed or fused glands is lower than that of pattern 4 with cribriform glands [18, 19]. The ISUP 2014 revision of the Gleason grading system suggested that there should be more than occasional examples of these structures present for the tumor to qualify as Gleason pattern 4 [6]. This is in line with a study that showed that for most uropathologists the presence of ≤ 5 seemingly poorly formed glands was not enough for a diagnosis of Gleason pattern 4 [20]. The Imagebase database allowed participants to classify cancers as bordering towards a lower or higher level. We have previously shown that among cases bordering between ISUP grades 1 and 2, the focus suggestive of the higher grade usually included seemingly poorly formed glands, while cribriform glands were not observed in any of these cases [3]. When the AI system classified the 13 non-consensus cases bordering between Gleason 3 + 3 and 3 + 4 with poorly formed or fused glands, it suggested the higher grade in 8 of them.

Another problem area is the estimation of the proportion of grades in a tumor that contains Gleason patterns 3 and 4. This is understandable as the patterns are often mixed and show a gradual transition. The problem appears in the separation between Gleason scores 3 + 4 and 4 + 3 and also between Gleason scores 4 + 3 and 4 + 4. We have previously shown that the lowest agreement among experts was reached in Gleason scores 4 + 3 and 4 + 4 [3]. In view of this, it is not surprising that the estimation of the extent of a minor component of Gleason pattern 3 is subjective. Cancers with different percentages of Gleason patterns 3 and 4 are currently reported as Gleason score 3 + 4, 4 + 3, or 4 + 4 (with less than < 5% pattern 3). It can be argued that the cutoff between 4 + 3 and 4 + 4 is unnecessary as the addition of a small component of pattern 3 in a tumor, which is otherwise composed of pattern 4, is unlikely to mitigate the aggressiveness of the cancer. Indeed, the outcome of Gleason score 4 + 3 and 4 + 4 cancers did not differ much in recent reports [21–24]. Furthermore, the number of cases assigned a Gleason score of 4 + 4 is very low in some series. For example, Sauter et al. assigned an ISUP grade 3 to 2236 out of 12,823 cancers (17.5%), while only 72 (0.6%) cases were considered ISUP grade 4 [24].

Among all cases, the weighted kappa of AI was the fourth lowest but it is remarkable that its performance was still within the range of the results of leading international experts (Fig. 2). Among the non-consensus cases the weighted kappa of AI was the sixth best (Fig. 2). This suggests that AI may assist in defining a standard in cases where pathologists struggle with the grading.

It can be argued that AI is not better equipped than an expert pathologist to assess where a line should be drawn between grades. Indeed, the AI decision is dependent on the environment in which it has been trained and it needs to be emphasized that AI has no deeper insight in the optimal grading than that provided by its training dataset. However, an advantage of AI is that it might be more consistent in its assessments and thereby bridge issues relating to inter-observer variability. Interestingly, the performance of the AI system was superior to that of most of the experts for the non-consensus cases. It is also of interest that the reproducibility of the AI system, relative to that of human observers, was greater in the non-consensus group than in the consensus group. A possible explanation is that the AI results were not included in the consensus decision and by definition the non-consensus group contained cases where human observers had failed to grade in a consistent manner, thus giving machine grading an advantage.

Despite an overall satisfactory performance, the AI system occasionally made grading decisions that deviated from the opinion of experts. In 6 cases, a grade was suggested that was not supported by any of the members of the expert panel. Particularly problematic were two cases of over-diagnosis of ISUP grade 5. This seems to be due to over-grading of occasional single cells that would be overlooked by expert pathologists. There is obviously still a requirement for the fine-tuning of the algorithms employed by the AI system in such cases. There is also a need to train the AI system in the grading of unusual morphological variants of prostate cancer. It seems that the greatest grading challenges are seen in high-grade cancers where there is a combination of patterns. The problem is not only to identify patterns but also to determine their proportions. Although the training set was enriched with high-grade cancers, it may be necessary to use an even greater number of cases of high complexity for training. The expectations on AI mechanisms are often unrealistic and we need to understand that their learning depends on huge training sets of high quality.

A limitation of this study is that the Imagebase panel graded microphotographs of cancers while the AI system used scanned slides of the same cases. The reason for not using scanned slides in Imagebase is that we aimed to set up a system that was easily accessible for pathologists across the globe, even in low resource areas. To enable a quick overview of multiple cases microphotographs are superior to scanned slides. It is also very challenging to make a group of leading international pathologists grade a large number of cancers. Doing this with scanned slides would have been even more time-consuming. On the other hand, microphotographs cannot be used for AI interpretation. Thus, the only way to carry out the study was to use these two technical platforms even though it limits the comparability.

One of the great challenges in pathology is to reduce the interobserver variability of assessments. Imagebase is an attempt to improve the reproducibility by setting up a catalog of cases for standardization of grading. AI could serve a similar purpose, ie. setting a standard for practicing pathologists by reducing subjectivity. It may also be used for external quality control. Even if AI misinterprets the grading of occasional cases, it would probably be useful to compare the grade distribution of a laboratory on a group level against the achievement of AI. Continuous interaction between human observers and machine learning has the potential to fine-tune not only the AI mechanism but also the subjective interpretation of pathologists.

It has been claimed that AI may reduce the workload of pathologists [25]. In relation to the reporting of prostate biopsies this could be achieved by reducing the assessment of benign biopsies and measuring cancer length automatically in positive biopsy cores. Whether this will be possible in clinical practice remains to be confirmed. In addition to its potential to provide primary assessments in the grading of prostate cancer, the AI system could also be utilized to provide second opinions, assist in standardizing grading and provide support and training in parts of the world where pathology expertise is not available [10]. However, it is important to understand that, at least for the present, AI will require supervision by a pathologist who is legally responsible for the final diagnosis. Further studies are required to determine how pathologists will manage results provided through AI support and how AI will, in the long term, influence subjective grading issues through the provision of continuous feedback.

Despite the progress in our understanding of the behavior of prostate cancer and the recognition of certain morphological landmark features, the grading of cancer is still to some extent an arbitrary segmentation of a continuous scale. The use of AI for setting the bar in borderline cases may assist in drawing meaningful biological boundaries in a more consistent manner. In the long term, it will be necessary to refine the AI tools by training them against large datasets with known outcome. There is, however, also a need to calibrate AI systems using data derived from studies that determine how morphology reflects the tumor biology. Through this, the results of genetic and clinical studies will be able to inform AI, thus permitting the delivery of more accurate education-based decisions.
